# Supplementary material for: Time‑resolved multi-omic analysis of paclitaxel exposure in human iPSC‑derived sensory neurons unveils mechanisms of chemotherapy‑induced peripheral neuropathy
Source: Cell Death Dis. 2026 Feb 10;17(1):211. doi: 10.1038/s41419-026-08445-2 (PMC12921266; doi:10.1038/s41419-026-08445-2)
Supplement: Supplementary file 15 — Dataset 1. [file 41419_2026_8445_MOESM15_ESM.zip › CDD_Open_Data/04_RNA_Seq/Timeline_panel_plots_etc_2023-01-23_1609.zip!/Panel_Plots_etc/Evidence_Plots/post5d/evidence_plot_standard contrasts_tmod_5d_PTX_vs_DMSO_ID6_DC.M3.2_inflammation.pdf]

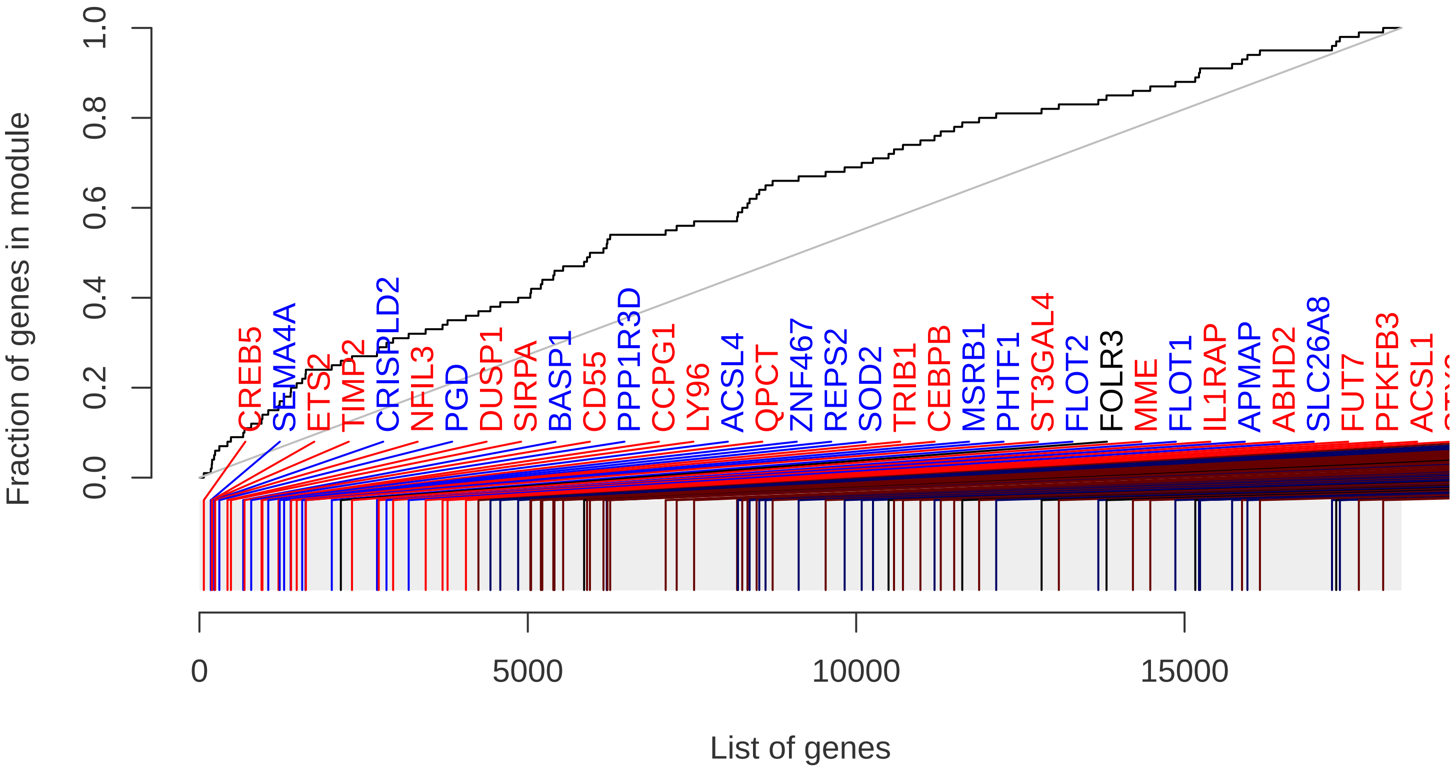

Fraction of genes in module
0.0
0.2
0.4
0.6
0.8
1.0
CREB5
SEMA4A
ETS2
TIMP2
CRISPLD2
NFIL3
PGD
DUSP1
SIRPA
BASP1
CD55
PPP1R3D
CCPG1
LY96
ACSL4
QPCT
ZNF467
REPS2
SOD2
TRIB1
CEBPB
MSRB1
PHTF1
ST3GAL4
FLOT2
FOLR3
MME
FLOT1
IL1RAP
APMAP
ABHD2
SLC26A8
FUT7
PFKFB3
ACSL1
0
5000
10000
15000
List of genes
